# Supplementary material for: Chromothripsis during telomere crisis is independent of NHEJ, and consistent with a replicative origin
Source: Genome Res. 2019 May;29(5):737–49. doi: 10.1101/gr.240705.118 (PMC6499312; doi:10.1101/gr.240705.118)
Supplement: Supplemental Material [file supp_gr.240705.118_Supplemental_file_1.zip › contigs/annotated_contigs/DB82/contig.3.DB82_length_609_mean_cov_6.89655172414.docx]

**DB82_length_609_mean_cov_6.89655172414**

```
CTAATGTAATTATTGAGGCATTGGTGACAATATTTCTTAGGGCCCTTCTAAGAAATATAGTGAGGGATGGACGAATAGAATGCATGGAT
 >chr5:165065159-165065476 + E=5e-176 p=3e-03
TAATCCAACATCCCTATTCTGTTAAACCTTTGGATTCTTTTCTGTACATTACAATGCAGGCTAATTGTAGGCTAATACTGAGTCCATGT

TTCAATCTATCAACCCAGAAAACCATGAGAACCGTGGCCATCTGTTCATGCTAACACCTTGAATTCAAAATCTCTGGTATGTGTATCAT

TATTGGTGGAATTAGACAAATTCTAACATAGCACTCTTGGAATCTGTGT|G|AGAGCTTTCAGGAAGAGCATGTCAAGAACAGAGATTT
                                                  >chr5:165066056-165066161 - E=2e-51
CAATTTTCAGCTCAAGATGTTGTCAGAAAACCATAGAACTTCTGTGATTACCCTAAAGGAGTATTCC|AAAGGTTTA|ACAGTTTGCAG
                                                                             >chr5:1650
CCTAGAATCCGGTGATTTCCTAGGAACTATTAATAGGTTCCTGCAGGATTTTTTTGCAAAATTTGAATCCATCGAACTGCATATTGTTG
66645-165066824 + E=5e-96
TTACAGTAACAAAACCATCACTTCTGTCTGCCCTTTACTTTTCAATCTAATTTATGTGACTTCCATAGTAGATGATAAG
```
